# Structural conditions required for the bridge lithiation and substitution of a basic calix[4]arene

**DOI:** 10.3762/bjoc.7.188

**Published:** 2011-11-30

**Authors:** Conrad Fischer, Wilhelm Seichter, Edwin Weber

**Affiliations:** 1 Institut für Organische Chemie, TU Bergakademie Freiberg, Leipziger Str. 29, 09596 Freiberg, Germany

**Keywords:** calixarene, lithiation, methylene bridge, supramolecular chemistry, X-ray structure

## Abstract

Lithiation and subsequent reaction with CO_2_ was applied to calix[4]arenes with different, equal or mixed, ether functions at the lower-rim site as well as *tert*-butylated or non-*tert-*butylated upper-rim positions. Whereas this reaction fails for symmetric calix[4]arene ethers with alkoxy residues greater than methoxy, the carboxylation of mixed methoxy-propoxy calixarene ethers is possible. In connection with this, several new monobridge-substituted calix[4]arenes were characterized with respect to their conformational behaviour in solution and the X-ray crystal structure of one key intermediate is taken into consideration.

## Introduction

**Scheme 1:**
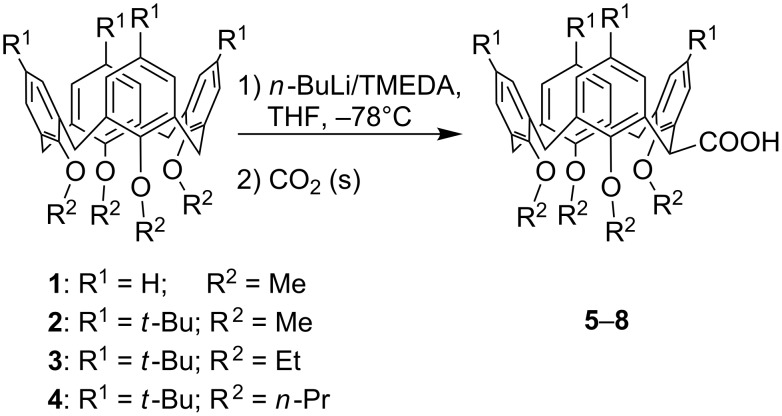
Calix[4]arene tetraethers **1**–**4** and corresponding bridge monosubstituted carboxylic acid derivatives **5**–**8**.

**Figure 1:**
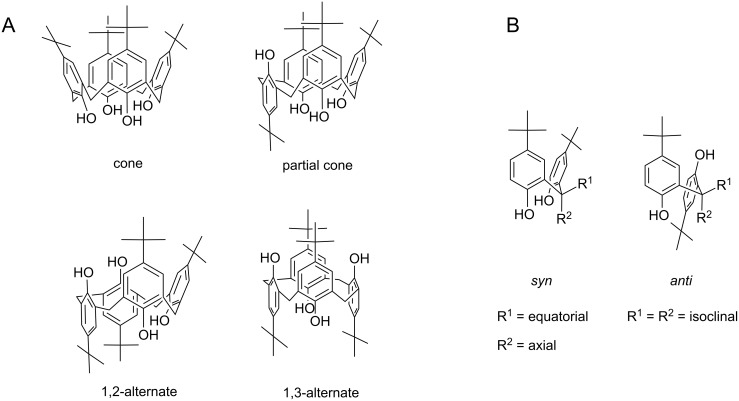
A: Four fundamental conformations of a calix[4]arene. B: Arrangement of the methylene group substituents with respect to the orientation of the neighbouring arene units.

Besides the huge progress made in the modification of the upper- and lower-rim positions of basic calix[4]arenes such as **1** in Scheme 1, the substitution of at least one methylene bridge of the chalice opens up a perspective for the vertical expansion of the molecule [1–2]. Thus, during the past decade two main preparative routes for the methylene-bridge substitution of *p*-*tert*-butyltetramethoxycalix[4]arene have been established: A protocol described by Biali et al. yields a stabilized methylene carbocation through bromination that is ready for electrophilic substitution under S_N_1 conditions [3], whereas we follow a route involving the formation of a methylene carbanion through lithiation, which by nucleophilic substitution forms the desired bridge-substituted calixarenes [4–5]. While the number of substituted methylene atoms in the first protocol depends clearly on the amount of *N*-bromosuccinimide used, surprisingly only monosubstitution of the chalice is observed by application of the lithiation technique, independent of the amount of *n*-BuLi. However, twofold substitution on opposite methylene bridges can be achieved by successive application of the latter technique [6]. This may suggest that the type of substitution significantly influences the conformation of the calixarene core, which is a versatile and important feature of this compound type (Figure 1). Nevertheless, in all these attempts for the purpose of a bridge substitution, only *p*-*tert*-butyltetramethoxycalix[4]arene **2** (Scheme 1) has been used as the basic compound. By way of contrast, nothing is known about the corresponding behaviour of upper-rim site unprotected or higher lower-rim site ether homologues as starting materials for a horizontal expansion of the chalice. Thus, within this paper, we present the results of the lithiation of differently upper- and lower-rim-modified calix[4]arenes, allowing us to draw helpful conclusions about the necessary structural requirements for the bridge lithiation and subsequent substitution of a basic calix[4]arene.

## Results and Discussion

Since the calixarene **2** is known to undergo lithiation and subsequent carboxylation to yield the carboxylic acid **6** [4], we became interested in the question of whether the upper-rim unsubstituted calix[4]arene **1**, as well as higher ether homologues, such as **3** and **4**, can also be used for this reaction (Scheme 1). While the upper-rim site unprotected calix[4]arene **1** can be similarly converted into the respective acid **5**, this reaction failed for the higher ether homologues **3** and **4**. In the case of **3**, only a few very weak signals in the ^1^H NMR spectrum suggested traces of the desired acid **7**, whereas upon addition of *n*-BuLi to a solution of the tetrapropyl ether **4** no colour change to cherry red (indicating the anion formation) was observed. Obviously, the lithiation step requires a high degree of conformational flexibility from the calixarene core, giving rise to coordinative interactions of the lithium cation with the methoxy O-atoms. For **1** and **2**, a rotation around the methylene groups is easily possible, thus allowing the conformational flexibility of the chalice, which is beneficial for the lithiation step. Though a rotation through the annulus may also take place in compound **3**, in principle, this conformational adaptation is slow on the timescale of lithiation. After addition of *n*-BuLi to a THF solution of the tetraethyl ether **3**, the colour changed over a period of 40 minutes only to strawberry red, indicating the highly limited reactivity of **3**. Since a rotation of any arene unit in the calix[4]arene **4** is impossible, due to the sterical hindrance of the propyl groups, no reaction with *n*-BuLi and CO_2_ was observed.

**Scheme 2:**
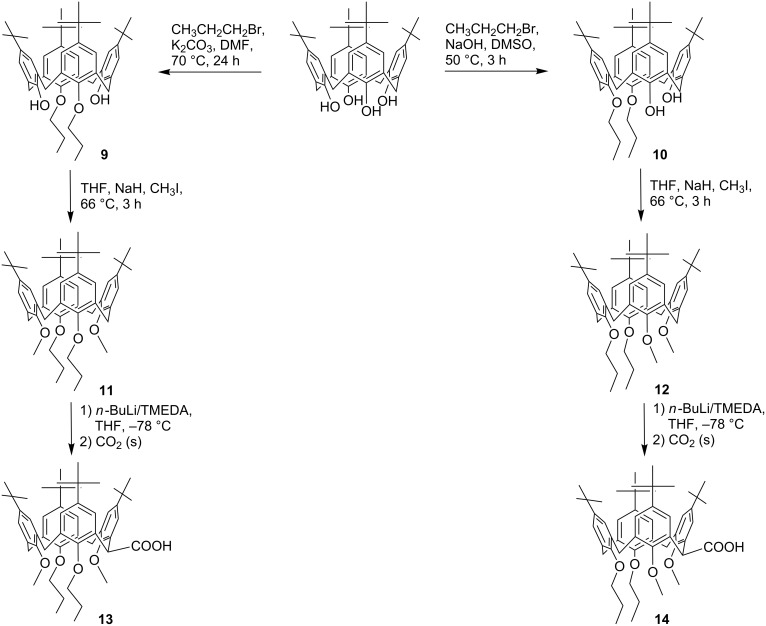
Pathways to the calixarene acids **13** and **14** bearing mixed ether functions in different fashions.

**Figure 2:**
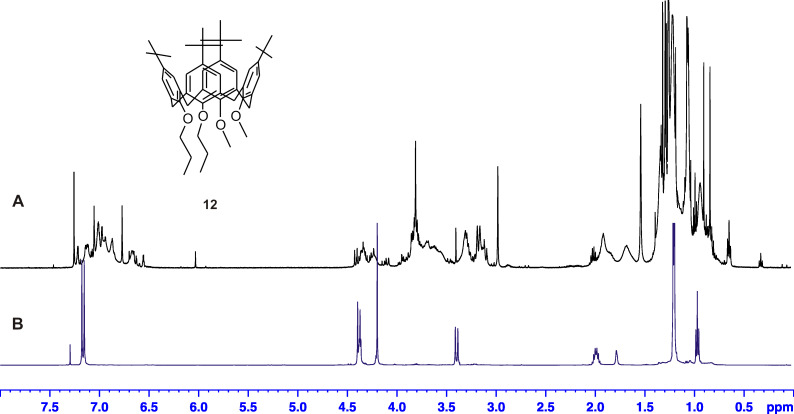
^1^H NMR spectrum (CDCl_3_, 293 K, 500 MHz) of calixarene ether **12** before (**A**) and after the addition of NaI/acetonitrile-*d*_3_ (**B**).

Considering the aforementioned facts, in a second attempt, we asked the question of whether the lithiation can also be achieved with mixed calix[4]arene ethers containing a combination of methoxy and propoxy groups at the lower-rim site. Therefore, the proximal and distal dipropoxy-dimethoxy ether derivatives **11** and **12** were synthesized in a two-step procedure by regioselective propylation of the parent tetrahydroxycalix[4]arene [7–8] and subsequent methylation of the intermediates **9** and **10** (Scheme 2). In accordance with a previous description [9], both derivatives show a high conformational flexibility in CDCl_3_ solution due to the possible rotation of the anisole units through the annulus, whereas the more bulky propoxy-containing arene units remain fixed. However, on the application of an elegant preparation procedure involving the addition of small amounts of NaI and acetronitrile-*d*_3_ to the CDCl_3_ solution, fixation of the calixarenes in a pure cone conformation was achieved (Figure 2).

Separate lithiation of the two mixed ethers **11** and **12** followed by the addition of CO_2_ results in the desired carboxylic acid derivatives **13** and **14**, which again feature high conformational flexibility in CDCl_3_ solution. Nevertheless, the yield of the acid **13** is much lower than for **14**, which is comprehensible since in compound **13** the lithium cation is always restricted by the steric demand of having one neighbouring propoxy group, limiting an ideal complexation. Again, on the addition of NaI and acetonitrile-*d*_3_ to the CDCl_3_ solution of **13** the cone conformation is preserved, bearing the lateral COOH group in an equatorial position between neighbouring methoxy and propoxy units, as indicated by NOESY interactions of the remaining methine proton. In contrast, after addition of the fixatives to a CDCl_3_ solution of **14**, a more complex signal pattern indicates the presence of at least two fixed conformations. One can be related to the expected resonances of the cone conformer, while the resonances of the second species fit best to a 1,2-alternate conformation (Supporting Information File 1, Figure S9). In both conformers, the lateral substituent is located at the equatorial position, assuming a rotation of both methoxy groups before the substitution step. Thus, a subsequent rotation of both methoxy groups through the annulus, transferring the 1,2-alternate to the cone conformer, is impossible, since it would ultimately lead to an axial orientation of the COOH group, which is strictly avoided for steric reasons [4,10].

### Crystal structure of compound 12

**Figure 3:**
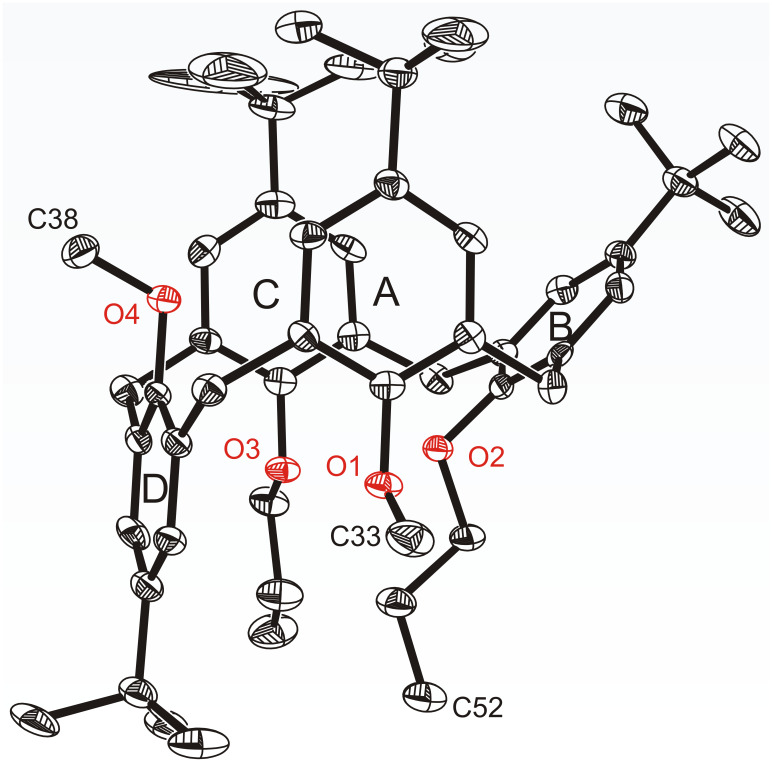
Crystal structure of compound **12**. For clarity only one of the two crystallographically independent molecules is presented; **12(1)** and H-atoms are omitted.

Crystallization of the mixed calixarene ether **12** from ethanol yielded colourless solvent-free crystals featuring two crystallographically independent molecules within the asymmetric unit (Figure 3). Unlike the findings relating to the conformational behaviour in solution, both structurally different calixarene molecules display a partial cone conformation with an anti-arrangement of the two neighbouring methoxy groups (Table 1). Whereas the molecule **12(1)** shows a nearly coplanar arrangement of the opposite arene rings A/C (4.12°), in the molecule **12(2)** these rings are inclined at an angle of 11.80°, which is attributable to packing effects. Remarkably, the two propyl chains seem to have no significant influence on the behaviour of the chalice with reference to related structures of tetramethoxycalix[4]arenes in a partial cone conformation [11–13].

**Table 1:** Selected conformational parameters of the mixed ether **12** in comparison with the corresponding tetramethoxycalix[4]arene **2**. Pairs (e.g. **12(1)** and **12(2)**) reflect two crystallographically independent molecules in these structures.

	12(1)	12(2)	2(1)	2(2)

Interplanar angle (°)				
A^a^/mpla^b^	86.21	79.86	88.27	86.20
B/mpla	44.87	42.39	35.35	36.28
C/mpla	89.68	88.36	84.82	84.74
D/mpla	85.17	77.62	88.00	88.39
A/C	4.12	11.80	6.96	9.09
B/D	40.31	35.29	52.65	52.18
KPI	62.9		–	

^a^Aromatic rings: A···C1–C6; B···C8–C13; C···C15–C20 and D···C22–C27. ^b^Mean plane through atoms C7, C14, C21 and C28.

In the packing (Supporting Information File 1, Figure S11), the molecules are stacked along the crystallographic *ab* plane stabilized by weak C–H···π interactions [14] involving methoxy groups and arene units of the neighbouring calixarene molecules (C33A–H33D···centroid *C*: 3.460(6) Å; C38–H38A···centroid *B*: 3.707(6) Å; C52A–H52D···centroid *A*: 3.619(6) Å). The resulting channel-like voids between any two calixarene layers are potentially accessible for guest inclusion, which is also reflected by a comparatively low packing index (62.9) of the crystal [15].

## Conclusion

It was shown that the bridge monosubstitution of a basic calix[4]arene via lithiation requires a high conformational flexibility of the calixarene, which is the case when the overall lower-rim substitution is not larger than methoxy and/or when the *tert*-butyl groups at the upper-rim site are absent. Moreover, in the case of mixed higher calixarene ethers bearing only two methoxy groups at the lower-rim site, the calixarene undergoes lithiation and substitution as well, indicating that the lithiation takes place favourably in the neighbourhood of the methoxy groups for potential complexation reasons. Once the bridge substitution has taken place in these types of calixarene ethers, a subsequent rotation around the methylene groups seems impossible, as suggested by the presence of two nonconvertible conformers of the calixarene acid **14**.

## Experimental

### General remarks

Melting points were determined on a microscope heating stage PHMK Rapido (VEB Dresden Analytik) and are uncorrected. IR spectra were measured on a Nicolet FT-IR 510 as KBr pellets. NMR spectra were recorded on a Bruker Avance DPX 500 spectrometer at 500.1 MHz (^1^H NMR) and 125.7 MHz (^13^C NMR), in CDCl_3_/CD_3_CN solution (9:1) with small amounts of NaI. Chemical shifts δ are reported in ppm relative to the internal reference TMS. Mass spectra were measured on a Varian 320 MS. Elemental analyses were perfomed on a Heraeus CHN-Rapid Analyzer. Reagents and chemicals for the synthesis were used as purchased from chemical suppliers. The solvents used were purified or dried according to common literature procedures.

### Syntheses

Starting compounds **1** [16], **2** [17], **3** [18] and **4** [7], as well as the intermediates **9** [7] and **10** [8] were prepared according to described protocols. The carboxylic acid **6** was prepared in a similar manner to compound **5**.

#### 25,26,27,28-Tetramethoxycalix[4]arene-2-carboxylic acid (**5**) (*cone*)

A solution of 1.7 mL (11.0 mmol) TMEDA in 100 mL dry THF was cooled down to −78 °C and at this temperature 5.2 mL (8.3 mmol) *n*-BuLi (1.6 M in *n*-hexane) was added. After 30 min a solution of 1.8 g (3.8 mmol) 25,26,27,28-tetramethoxycalix[4]arene (**1**) in 50 ml dry THF was added by syringe. The resulting cherry red solution was allowed to warm up to ambient temperature. After 1 h, solid CO_2_ was added, immediately changing the colour of the solution to yellow. Removal of all volatiles resulted in a yellow residue, which was dissolved in a small amount of methanol. By addition of water, a white precipitate was formed giving **5** after recrystallization from methanol as a microcrystalline solid. Yield: 0.9 g (46%); mp 249–251 °C; ^1^H NMR (500.1 MHz, CDCl_3_/CD_3_CN 9:1) δ 7.44 (d, *J* = 7.5 Hz, 2H, ArH), 7.19 (d, *J* = 7.5 Hz, 2H, ArH), 7.15 (d, *J* = 7.5 Hz, 4H, ArH), 6.96 (t, *J* = 7.8 Hz, 2H, ArH), 6.90 (t, *J* = 7.8 Hz, 2H, ArH), 5.83 (s, 1H, CHCOOH), 4.33 (m, 4H, ArCH_2_Ar), 4.27 (s, 6H, OCH_3_), 4.22 (s, 6H, OCH_3_), 3.48 (m, 4H, ArCH_2_Ar); ^13^C NMR (125.7 MHz, CDCl_3_/CD_3_CN 9:1) δ 177.8 (COOH), 152.7, 152.4, 134.9, 134.8, 134.7, 129.8, 129.2, 129.0, 127.6, 126.2, 126.0, 65.7, 65.1, 40.8, 29.4; IR (cm^−1^): 2931, 2820, 1714, 1588, 1463, 1426, 1326, 1288, 1248, 1206, 1168, 1087, 1009, 897, 838, 771, 685, 612; LC–MS *m*/*z*: 542.2 [M + NH_4_]^+^; Anal. calcd for C_33_H_32_O_6_: C, 71.85; H, 6.40; found: C, 72.00; H, 6.28.

#### General procedure for the synthesis of **11** and **12** (*O*-methylation)

The given amount of distal and proximal *O*-propoxylated calix[4]arenes **9** and **10** was dissolved in dry THF and NaH (60% in mineral oil) was added. After 1.5 h of reflux, 9 equiv of MeI were added by syringe and heating was continued for 3 h. All volatiles were removed and the crude product was treated carefully with water. The solid was separated and dissolved in 30 mL CHCl_3_. After addition of MeOH, a white precipitate formed, which was collected and recrystallized from CHCl_3_/MeOH 1:3.

#### 5,11,17,23-Tetra-*tert-*butyl-25,27-dimethoxy-26,28-dipropoxycalix[4]arene (**11**) (*cone*)

Reagents: 2.85 g (3.9 mmol) **9** in 40 mL dry THF, 1.6 g (40 mmol) NaH, 2.3 mL (5.1 g, 36 mmol) CH_3_I. Yield: 1.7 g (58%); mp 203–206 °C; ^1^H NMR (500.1 MHz, CDCl_3_/CD_3_CN 9:1) δ 7.18 (s, 4H, ArH), 7.15 (s, 4H, ArH), 4.38 (d, *J* = 12.4 Hz, 4H, ArCH_2_Ar), 4.37 (m, 4H, C*H*_2_CH_2_CH_3_), 4.20 (s, 6H, OCH_3_), 3.39 (d, *J* = 12.4 Hz, 4H, ArCH_2_Ar), 1.99 (m, 4H, CH_2_C*H*_2_CH_3_), 1.21 (s, 18H, C(CH_3_)_3_), 1.20 (s, 18H, C(CH_3_)_3_), 0.97 (t, *J* = 7.4 Hz, 6H, CH_2_CH_2_C*H*_3_); ^13^C NMR (125.7 MHz, CDCl_3_/CD_3_CN 9:1) δ 150.8, 149.9, 148.7, 148.0, 134.8, 134.6, 125.8, 125.7, 79.7, 64.7, 34.2, 34.1, 31.1, 31.0, 30.4, 22.6, 9.3; LC–MS *m*/*z*: 778.6 [M + NH_4_]^+^; Anal. calcd for C_52_H_72_O_4_·CH_3_OH: C, 81.14; H, 9.59; found: C, 81.01; H, 9.38.

#### 5,11,17,23-Tetra-*tert*-butyl-25,26-dimethoxy-27,28-dipropoxycalix[4]arene (**12**) (*cone*)

Reagents: 2.2 g (3.0 mmol) **10** in 40 mL dry THF, 1.2 g (30 mmol) NaH, 1.7 mL (3.8 g, 27 mmol) CH_3_I. Yield: 1.4 g (61%); mp 154–156 °C; ^1^H NMR (500.1 MHz, CDCl_3_/CD_3_CN 9:1) δ 7.17 (s, 4H, ArH), 7.17 (s, 2H, ArH), 7.16 (s, 2H, ArH), 4.47 (d, *J* = 12.4 Hz, 1H, ArCH_2_Ar), 4.39 (d, *J* = 12.4 Hz, 2H, ArCH_2_Ar), 4.35 (m, 4H, C*H*_2_CH_2_CH_3_), 4.29 (d, *J* = 12.4 Hz, 1H, ArCH_2_Ar), 4.21 (s, 6H, OCH_3_), 3.40 (m, 4H, ArCH_2_Ar), 2.00 (m, 4H, CH_2_C*H*_2_CH_3_), 1.21 (s, 18H, C(CH_3_)_3_), 1.20 (s, 18H, C(CH_3_)_3_), 0.97 (t, *J* = 7.4 Hz, 6H, CH_2_CH_2_C*H*_3_); ^13^C NMR (125.7 MHz, CDCl_3_/CD_3_CN 9:1) δ 150.8, 149.8, 148.6, 148.1, 135.0, 134.7, 134.6, 134.4, 125.9, 125.8, 125.7, 125.6, 79.6, 65.0, 34.2, 34.1, 31.0, 31.0, 30.4, 22.5, 9.3; LC–MS *m*/*z*: 778.6 [M + NH_4_]^+^; Anal. calcd for C_52_H_72_O_4_: C, 82.06; H, 9.53; found: C, 82.22; H, 9.67.

#### 5,11,17,23-Tetra*-tert*-butyl-25,27-dimethoxy-26,28-dipropoxycalix[4]arene-2-carboxylic acid (**13**) (*cone*)

Lithiation and subsequent substitution followed the described protocol for compound **5**. Reagents: 0.76 g (1 mmol) mixed ether **11** in 25 mL dry THF, 0.95 mL (6.1 mmol) TMEDA in 40 mL dry THF, 2.85 mL (4.6 mmol) *n*-BuLi. Yield: 0.28 g (36%); mp 147–150 °C; ^1^H NMR (500.1 MHz, CDCl_3_/CD_3_CN 9:1) δ 7.16 (d, *J* = 12 Hz, 6H, ArH), 7.06 (d, *J* = 5 Hz, 2H, ArH), 5.86 (s, CHCOOH), 4.38 (d, *J* = 12.5 Hz, 3H, ArCH_2_Ar), 4.37 (m, 4H, C*H*_2_CH_2_CH_3_), 4.20 (s, 6H, OCH_3_), 3.39 (d, *J* = 12.5 Hz, 3H, ArCH_2_Ar), 1.99 (m, 4H, CH_2_C*H*_2_CH_3_), 1.21 (s, 18H, C(CH_3_)_3_), 1.20 (s, 18H, C(CH_3_)_3_), 0.97 (t, *J* = 7.3 Hz, 6H, CH_2_CH_2_C*H*_3_); ^13^C NMR (125.7 MHz, CDCl_3_/CD_3_CN 9:1) δ 179.3 (COOH), 150.8, 150.0, 148.7, 148.1, 134.8, 134.6, 133.7, 132.5, 132.1, 127.8, 125.8, 125.7, 125.5, 125.4, 79.7, 64.8, 64.6, 34.2, 34.1, 31.5, 31.4, 31.1, 30.9, 30.4, 22.6, 9.3; IR (cm^−1^): 2959, 2905, 2873, 2822, 1707, 1602, 1481, 1462, 1391, 1296, 1245, 1202, 1122, 1067, 1043, 1013, 966, 947, 870, 815, 698, 640, 556; LC–MS *m*/*z*: 822.6 [M + NH_4_]^+^; Anal. calcd for C_53_H_72_O_6_: C, 79.06; H, 9.01; found: C, 79.12; H, 9.21.

#### 5,11,17,23-Tetra-*tert*-butyl-25,26-dimethoxy-27,28-dipropoxycalix[4]arene-2-carboxylic acid (**14**)

Lithiation and subsequent substitution followed the described protocol for compound **5**. Reagents: 0.71 g (0.9 mmol) mixed ether **12** in 25 mL dry THF, 0.95 g (6.1 mmol) TMEDA in 40 mL dry THF, 2.85 mL (4.6 mmol) *n*-BuLi. Yield: 0.45 g (60%); mp 130–133 °C; ^1^H NMR (500.1 MHz, CDCl_3_/CD_3_CN 9:1) δ 7.17 (m, 8H, ArH), 5.86 (s, 1H, CHCOOH), 4.53 (d, *J* = 12 Hz, 1H, ArCH_2_Ar), 4.43 (d, *J* = 12.5 Hz, 2H, ArCH_2_Ar), 4.35 (m, 4H, C*H*_2_CH_2_CH_3_), 4.27 (s, 3H, OCH_3_), 4.20 (s, 3H, OCH_3_), 3.42 (m, 3H, ArCH_2_Ar), 2.00 (m, 4H, CH_2_C*H*_2_CH_3_), 1.21 (s, 18H, C(CH_3_)_3_), 1.20 (s, 18H, C(CH_3_)_3_), 0.96 (t, *J* = 7.3 Hz, 6H, CH_2_CH_2_C*H*_3_); ^13^C NMR (125.7 MHz, CDCl_3_/CD_3_CN 9:1) δ 173.9 (COOH), 153.4, 150.6, 150.3, 149.7, 148.7, 134.8, 134.6, 134.2, 133.8, 132.8, 132.6, 132.1, 126.7, 126.4, 126.1, 125.7, 125.5, 79.9, 79.5, 65.5, 65.0, 60.5, 41.3, 41.0, 37.2, 34.3, 34.0, 33.8, 31.4, 31.3, 31.0, 29.6, 22.5, 22.2, 9.9, 9.2; IR (cm^−1^): 2955, 2904, 2871, 2820, 1707, 1602, 1481, 1463, 1391, 1361, 1287, 1245, 1201, 1121, 1067, 1043, 1018, 967, 948, 869, 789, 677, 637, 554; LC–MS *m*/*z*: 822.6 [M + NH_4_]^+^; Anal. calcd for C_53_H_72_O_6_: C, 79.06; H, 9.01; found: C, 78.75; H, 9.03.

### Crystallography

The intensity data for the crystals of compound **12** submitted to X-ray diffraction were collected on a Kappa APEX II diffractometer (Bruker-AXS) with graphite-monochromated Mo Kα radiation (λ = 0.71073 Å) using ω- and φ-scans. Reflections were corrected for background, Lorentz and polarization effects. Preliminary structure models were derived by application of direct methods [19] and were refined by full-matrix least-squares calculations based on *F*^2^ for all reflections. An empirical absorption correction based on multiple scans was applied by using the SADABS program [20]. All non-hydrogen atoms were refined anisotropically. All hydrogen atoms were refined as being constrained to bonding atoms.

**Crystal data for 12:** C_52_H_72_O_4_, crystal system, space group: Monoclinic, *P*2_1_/*c*; unit cell dimensions: *a* = 17.3537 (8) Å, *b* = 20.1153 (9) Å, *c* = 27.2570 (13) Å, β = 91.930° (2); volume: 9509.3 (8) Å^3^; Z = 8; calculated density: 1.063 mg m^−3^; absorption coefficient: 0.065; *F*(000): 3328; θ-range for data collection 1.17–25.03°; refinement method: Full matrix least-square on F^2^; data/parameters: 7791/1041; goodness-of-fit on *F*^2^: 1.013; final *R* indices [*I* >2 σ(*I*)]: *R* = 0.0944, *wR* = 0.1970; *R*-indices (all data): *R* = 0.2010, *wR* = 0.2517; final Δρ_max_/Δρ_min_: 0.49/−0.45 *e* Å^−3^. CCDC: 847217.

## Supporting Information

File 1 ^1^H NMR and ^13^C NMR spectra of compounds **5**, **11**–**14** and crystal packing illustration of mixed ether **12**.

## References

[1] Fischer C, Gruber T, Seichter W, Weber E (2011). Org Biomol Chem.

[2] Fischer C, Lin G, Bombicz P, Seichter W, Weber E (2011). Struct Chem.

[3] Kuno L, Biali S E (2011). J Org Chem.

[4] Gruber T, Gruner M, Fischer C, Seichter W, Bombicz P, Weber E (2010). New J Chem.

[5] Gruner M, Fischer C, Gruber T, Weber E (2010). Supramol Chem.

[6] Fischer C, Lin G, Seichter W, Weber E (2011). Tetrahedron.

[7] Iwamoto K, Araki S, Shinkai S (1991). J Org Chem.

[8] Boyko V I, Podoprigorina A A, Yakovenko A V, Pirozhenko V V, Kalchenko V I (2004). J Inclusion Phenom Macrocyclic Chem.

[9] Matthews S E, Saadioui M, Böhmer V, Barboso S, Arnaud-Neu F, Schwing-Weill M-J, Carrera A G, Dozol J-F (1999). J Prakt Chem.

[10] Biali S E, Böhmer V, Cohen S, Ferguson G, Grüttner C, Grynszpan F, Paulus E F, Thondorf I, Vogt W (1996). J Am Chem Soc.

[11] Grootenhuis P D J, Kollman P A, Groenen L C, Reinhoudt D N, Van Hummel G J, Ugozzoli F, Andreetti G D (1990). J Am Chem Soc.

[12] Fischer C, Gruber T, Seichter W, Weber E (2007). Acta Crystallogr.

[13] Fischer C, Gruber T, Seichter W, Schindler D, Weber E (2008). Acta Crystallogr.

[14] Nishio M, Umezawa Y, Honda K, Tsuboyama S, Suezawa H (2009). CrystEngComm.

[15] Kitaigorodskii A L (1973). Molecular Crystals and Molecules.

[16] Harada T, Rudziński J M, Shinkai S (1992). J Chem Soc, Perkin Trans 2.

[17] Gutsche C D, Dhawan B, Levine J A, No K H, Bauer L J (1983). Tetrahedron.

[18] Araki K, Iwamoto K, Shinkai S, Matsuda T (1989). Chem Lett.

[19] Sheldrick G M (2008). Acta Crystallogr.

[20] (2004). SADABS.

