# Supplementary material for: Structural conditions required for the bridge lithiation and substitution of a basic calix[4]arene
Source: Beilstein J Org Chem. 2011 Nov 30;7:1602–8. doi: 10.3762/bjoc.7.188 (PMC3252863; doi:10.3762/bjoc.7.188)
Supplement: File 1 — 1H NMR and 13C NMR spectra of compounds 5, 11–14 and crystal packing illustration of mixed ether 12. [file Beilstein_J_Org_Chem-07-1602-s001.pdf]

Supporting Information

for

Structural conditions required for the bridge lithiation and substitution of a basic calix[4]arene

Conrad Fischer, Wilhelm Seichter and Edwin Weber*

Address: Institut für Organische Chemie, TU Bergakademie Freiberg, Leipziger Str. 29, 09596 Freiberg, Germany

Email: Edwin Weber - Edwin.Weber@chemie.tu-freiberg.de

* Corresponding author

^{1}H NMR and ^{13}C NMR spectra of compounds 5, 11–14 and crystal packing illustration of mixed ether 12.

^{1}H NMR and ^{13}C NMR spectra of compounds **5**, **11**, **12**, **13** and **14** S2–S11

Crystal packing illustration of mixed ether **12** S12

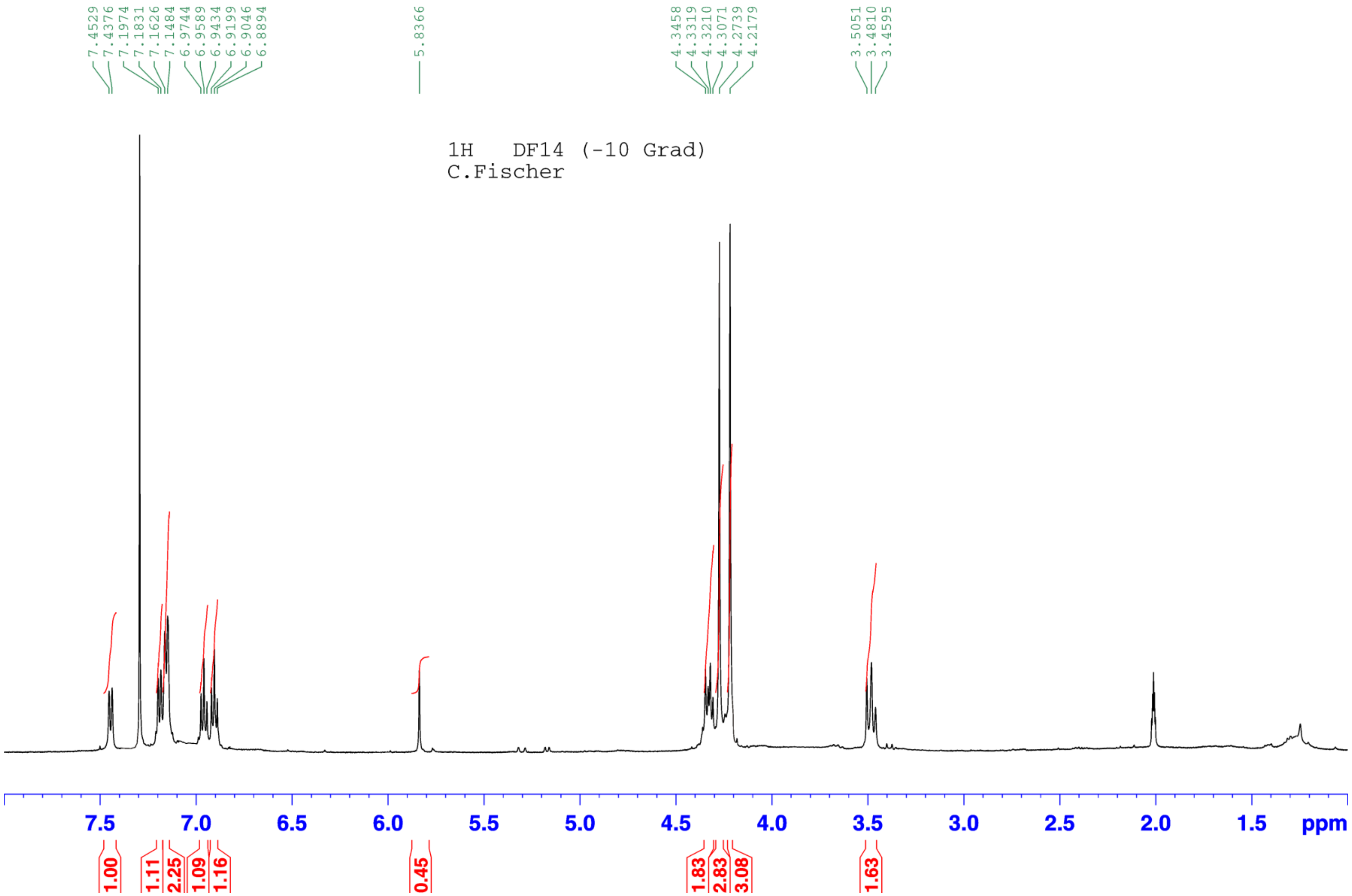

Figure S1: ^{1}H NMR spectrum of **5** in $CDCl_3$ (incl. NaI/acetonitrile-d_3) at 263 K.

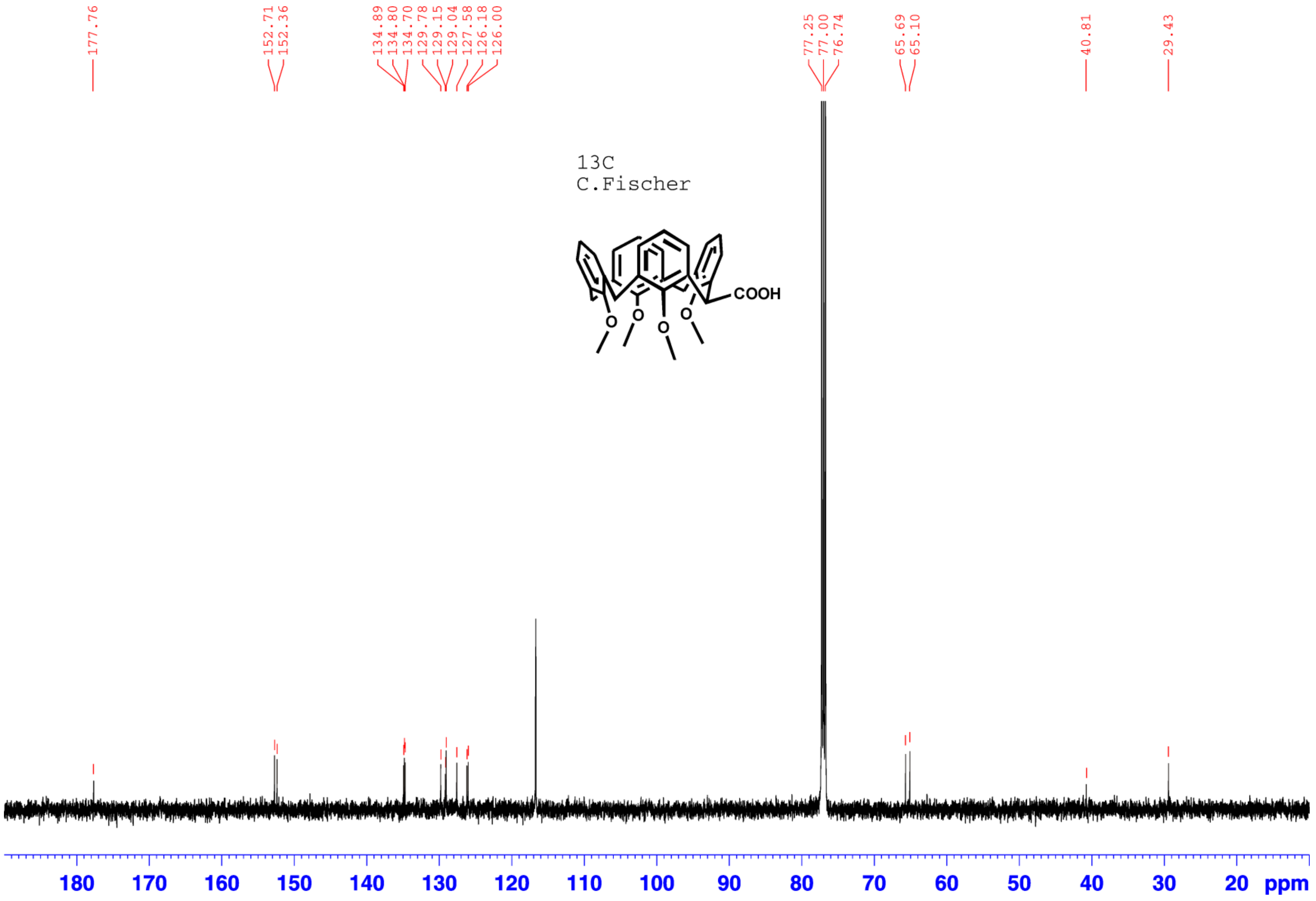

Figure S2: ^{13}C NMR spectrum of **5** in $CDCl_3$ (incl. NaI/acetonitrile-d_3) at 263 K.

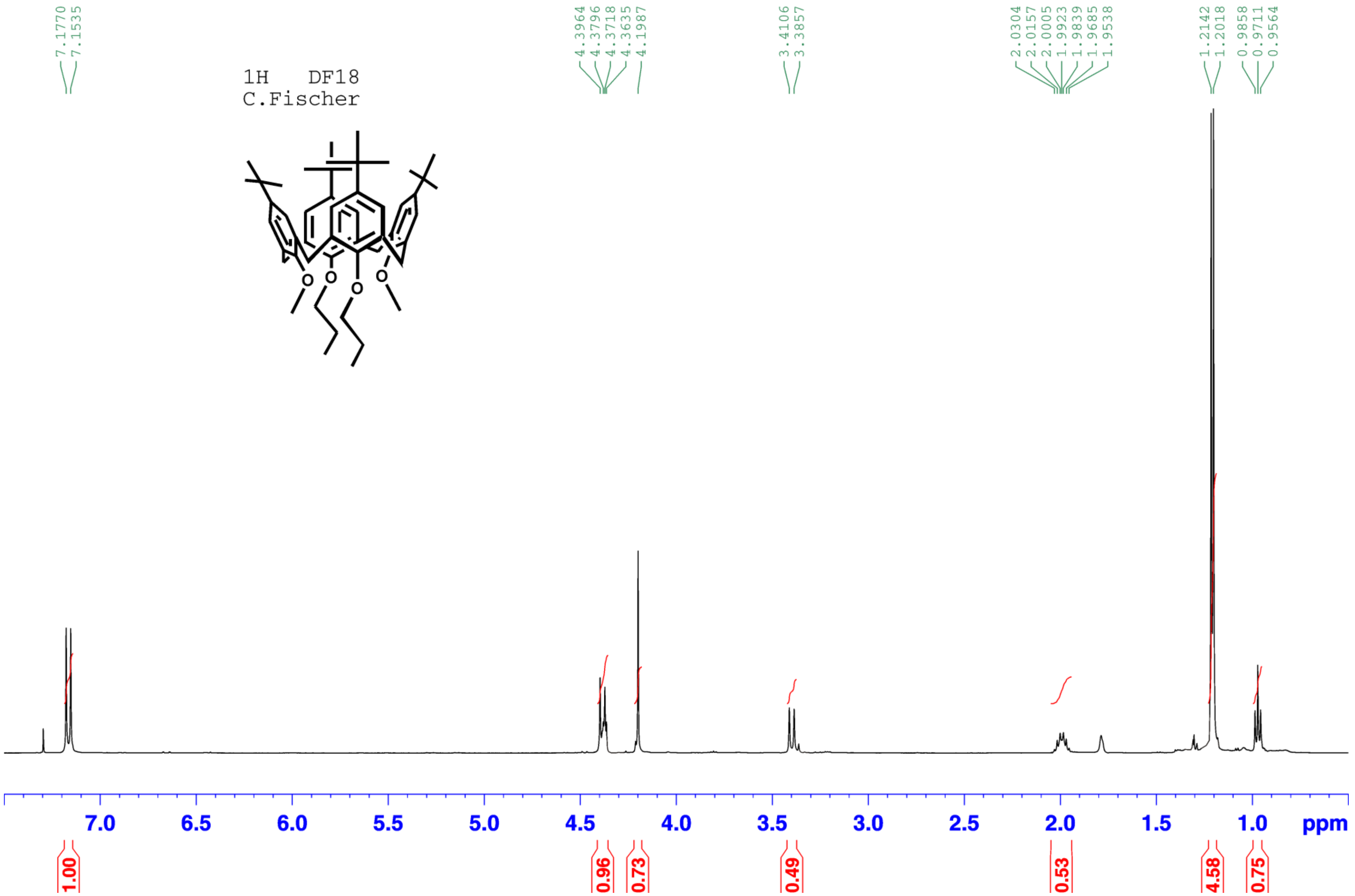

Figure S3: ^{1}H NMR spectrum of **11** in $CDCl_3$ (incl. NaI/acetonitrile-d_3) at 293 K.

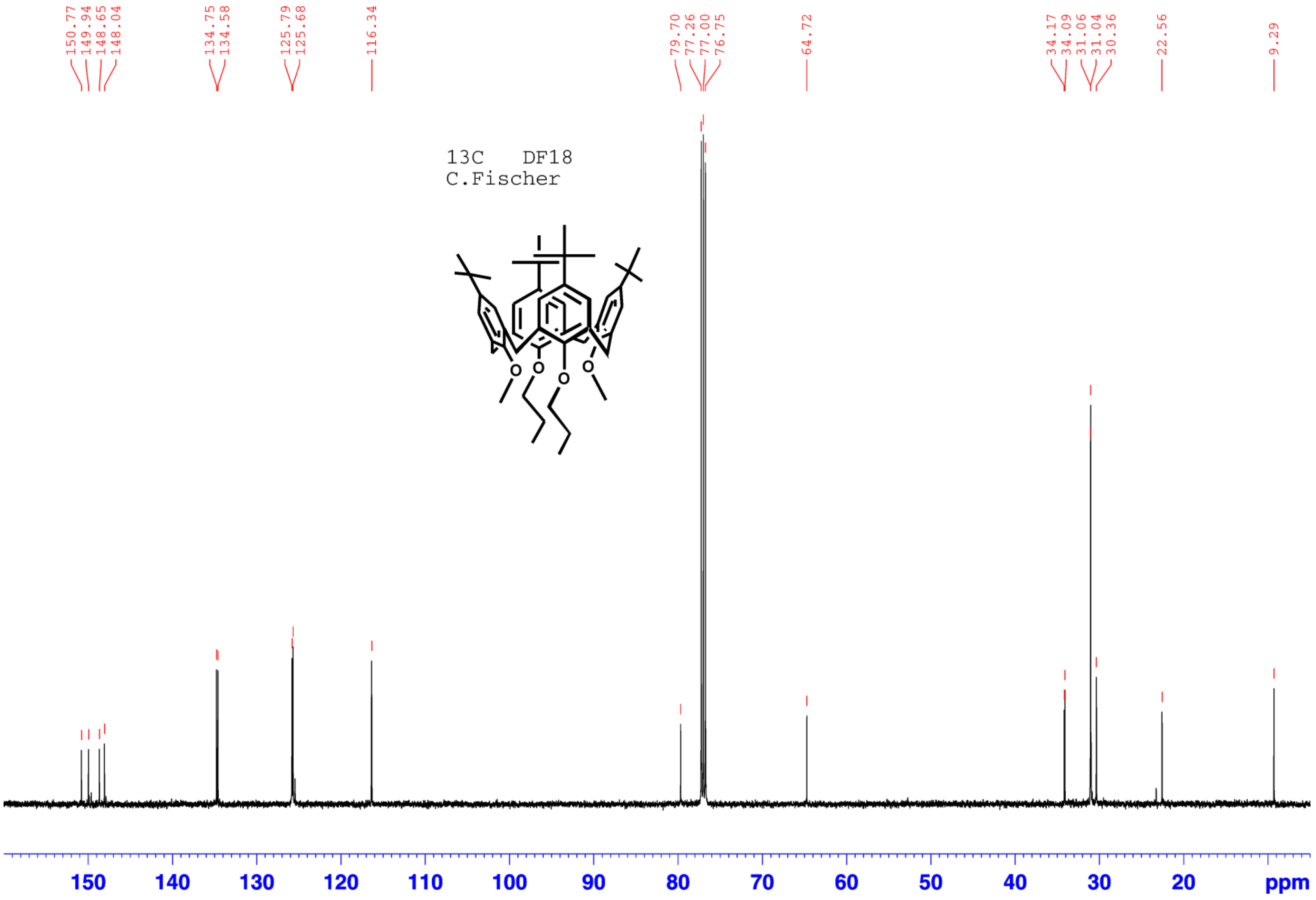

Figure S4: ^{13}C NMR spectrum of **11** in $CDCl_3$ (incl. NaI/acetonitrile-d_3) at 293 K.

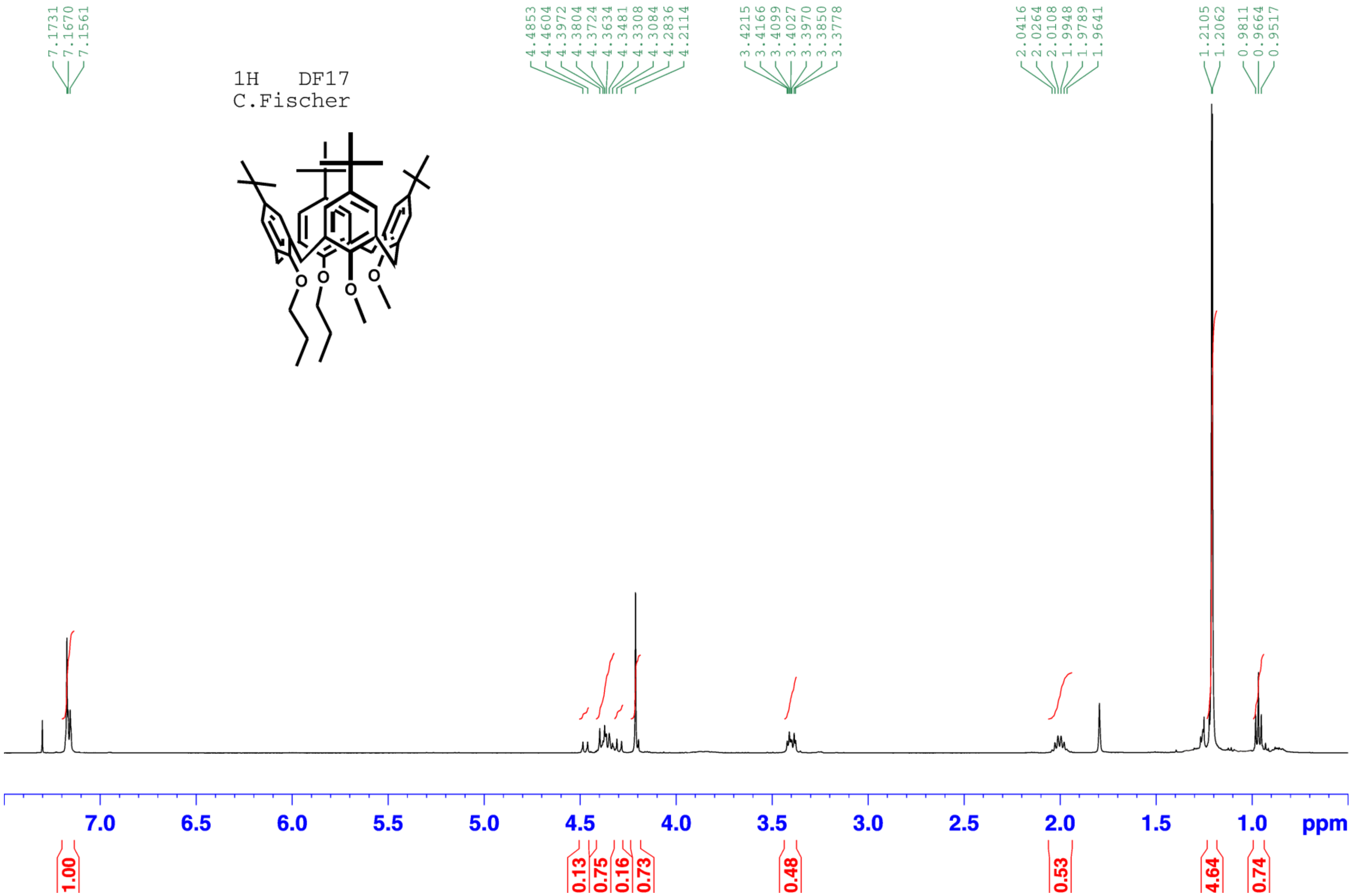

Figure S5: ^{1}H NMR spectrum of **12** in $CDCl_3$ (incl. NaI/acetonitrile-d_3) at 293 K.

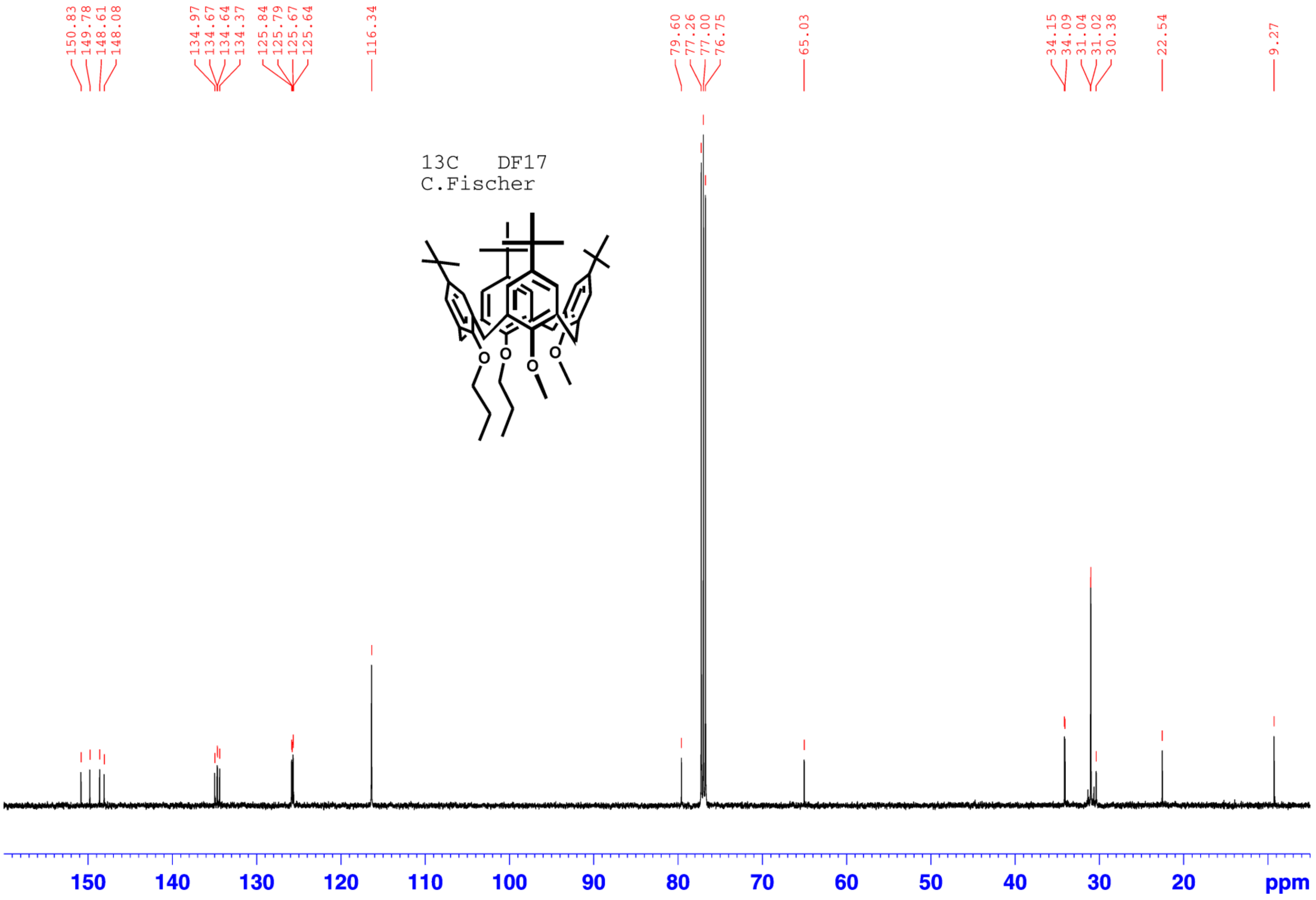

Figure S6: ^{13}C NMR spectrum of **12** in $CDCl_3$ (incl. NaI/acetonitrile-d_3) at 293 K.

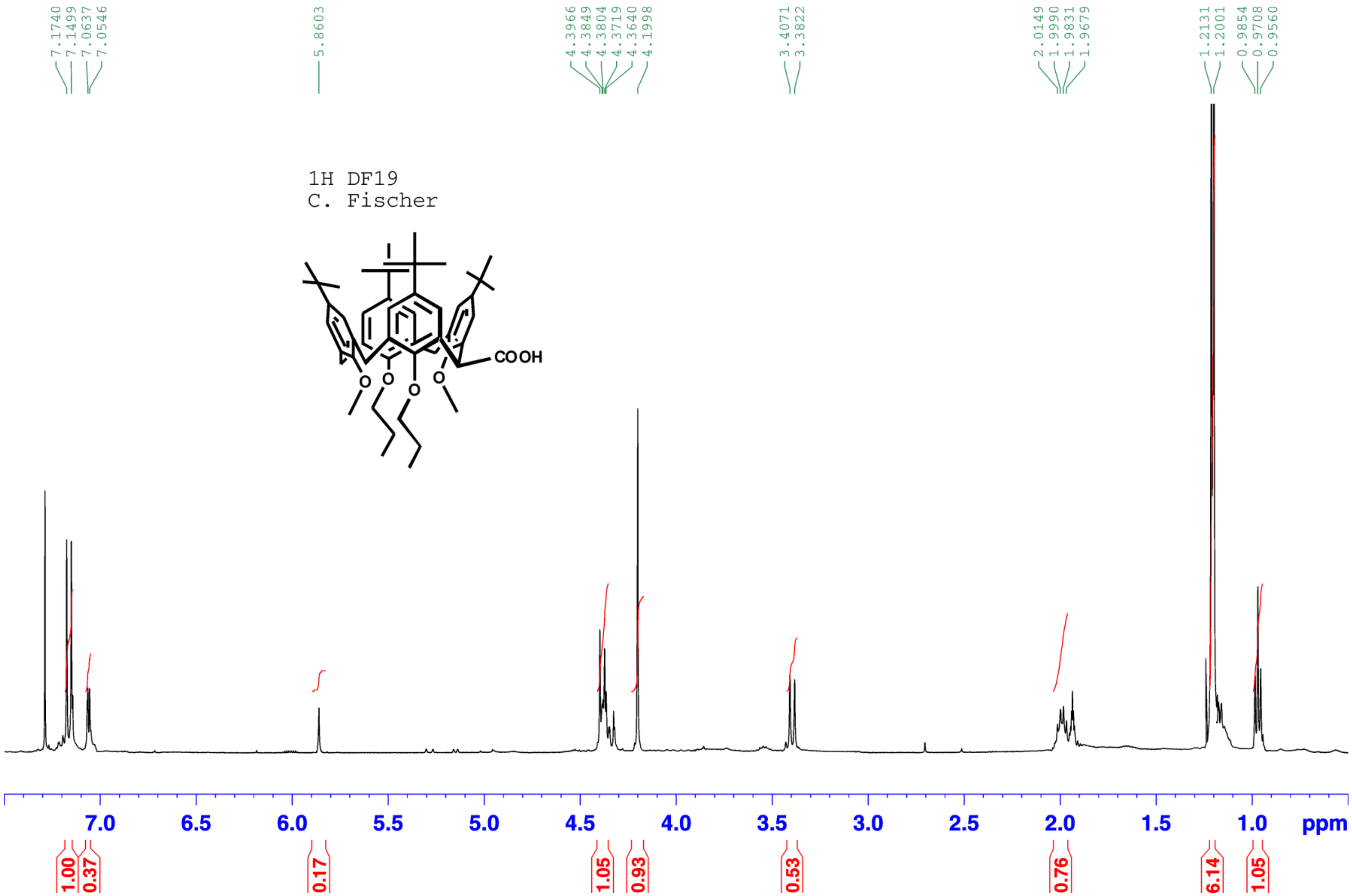

Figure S7: ^{1}H NMR spectrum of **13** in $CDCl_3$ (incl. NaI/acetonitrile-d_3) at 293 K.

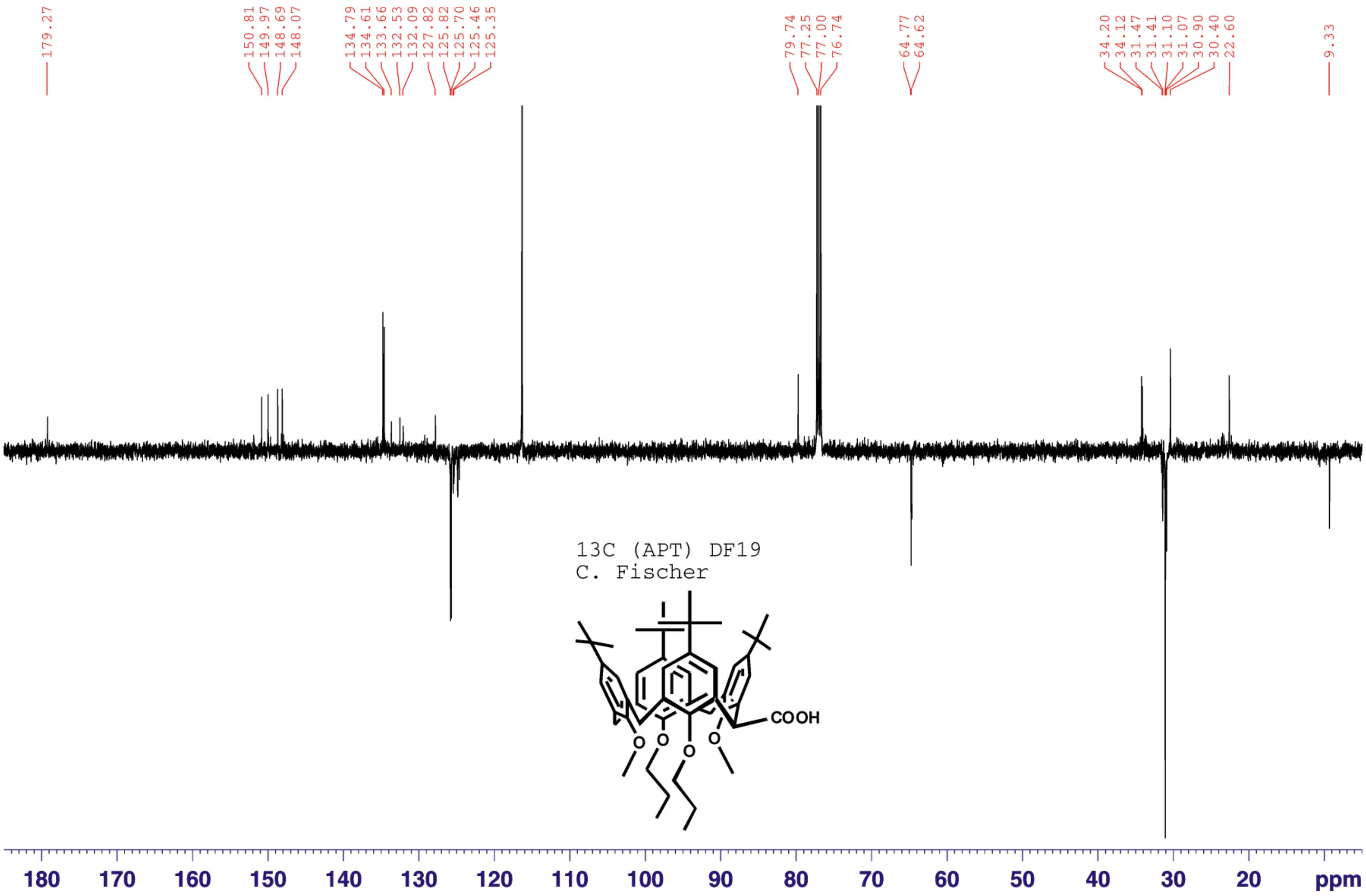

Figure S8: ^{13}C APT NMR spectrum of **13** in $CDCl_3$ (incl. NaI/acetonitrile-d_3) at 293 K.

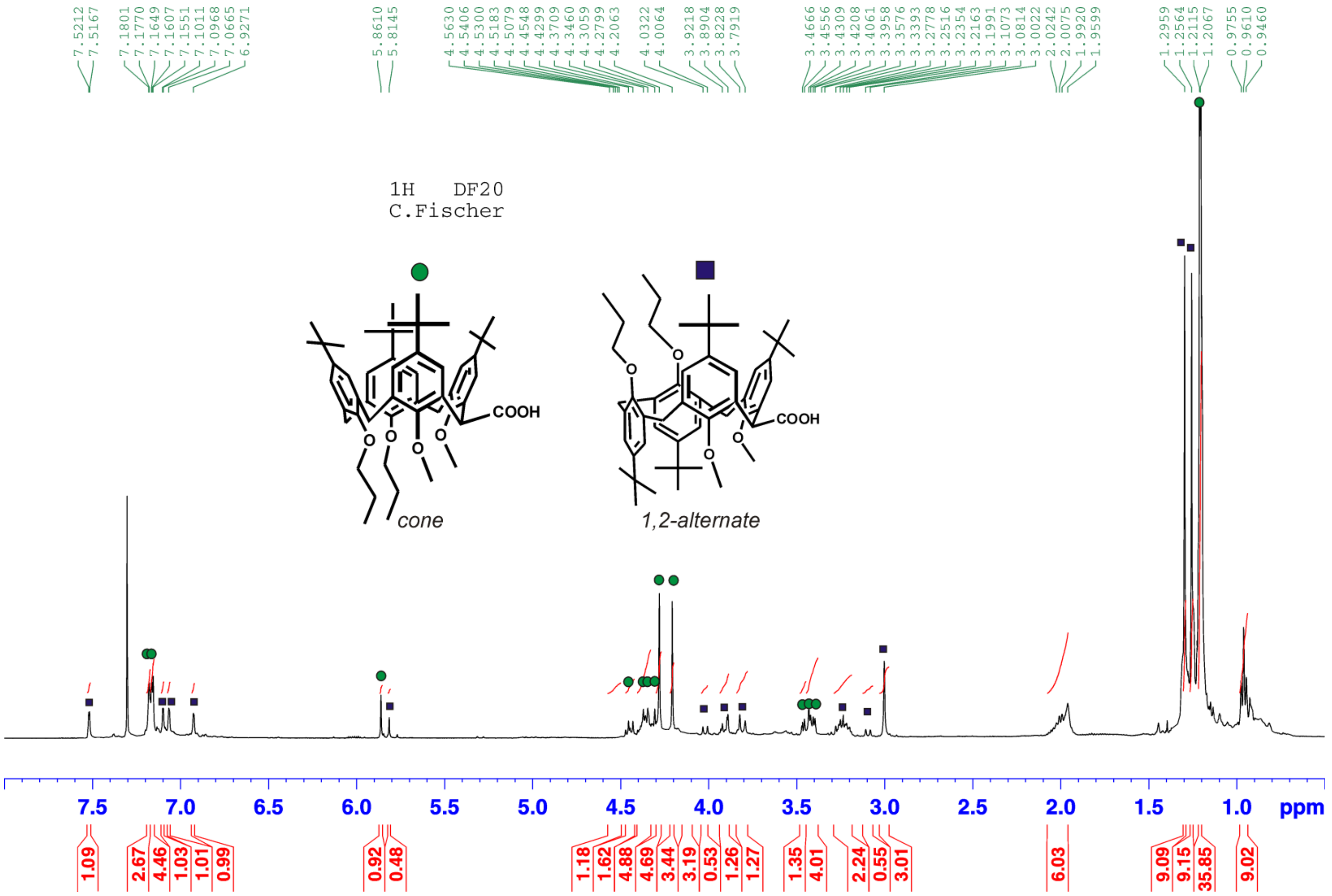

Figure S9: ^{1}H NMR spectrum of **14** in $CDCl_3$ (incl. NaI/acetonitrile-d_3) at 293 K with assignment of the resonances of the two different conformers.

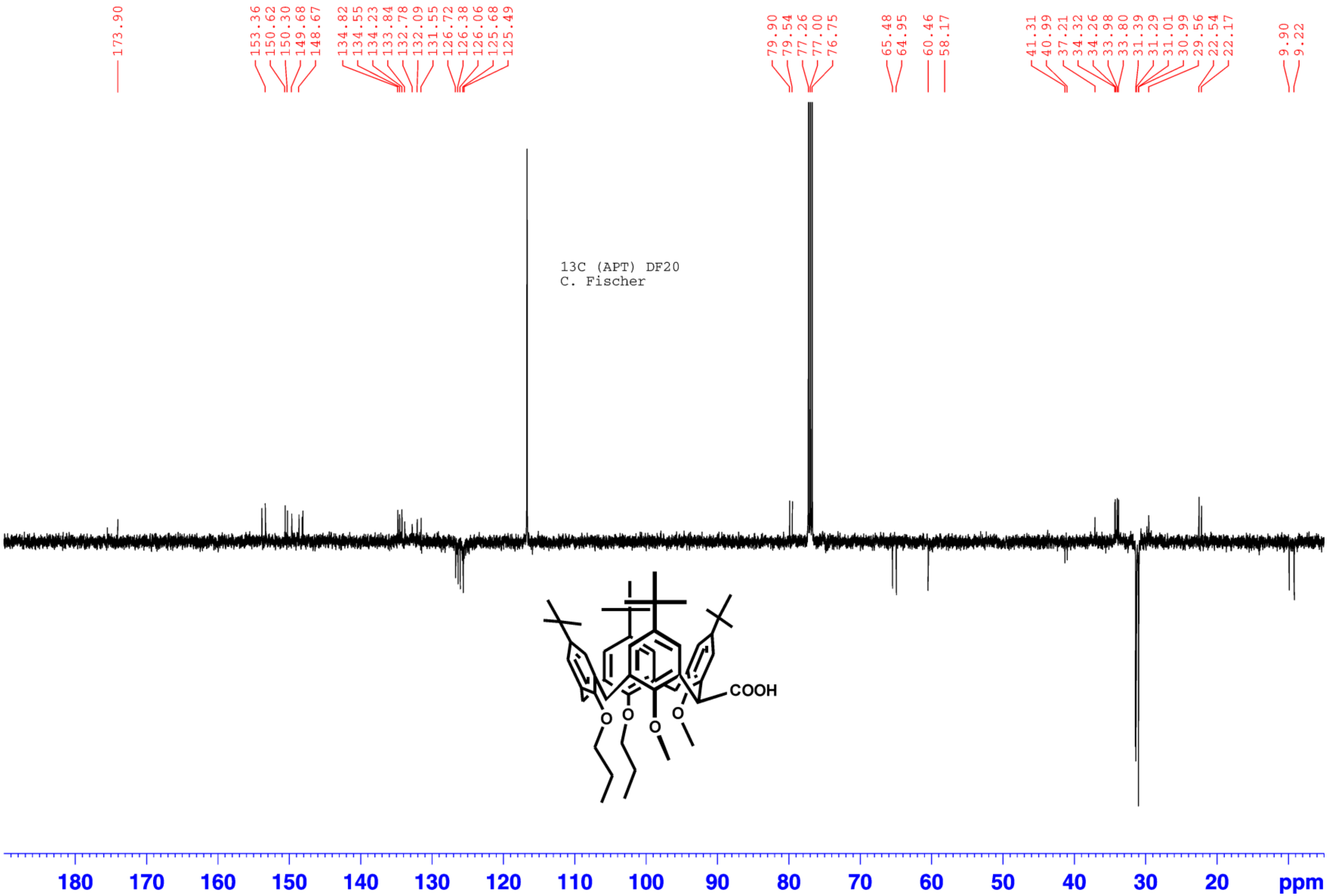

Figure S10: ^{13}C APT NMR spectrum of **14** in $CDCl_3$ (incl. NaI/acetonitrile-d_3) at 293 K.

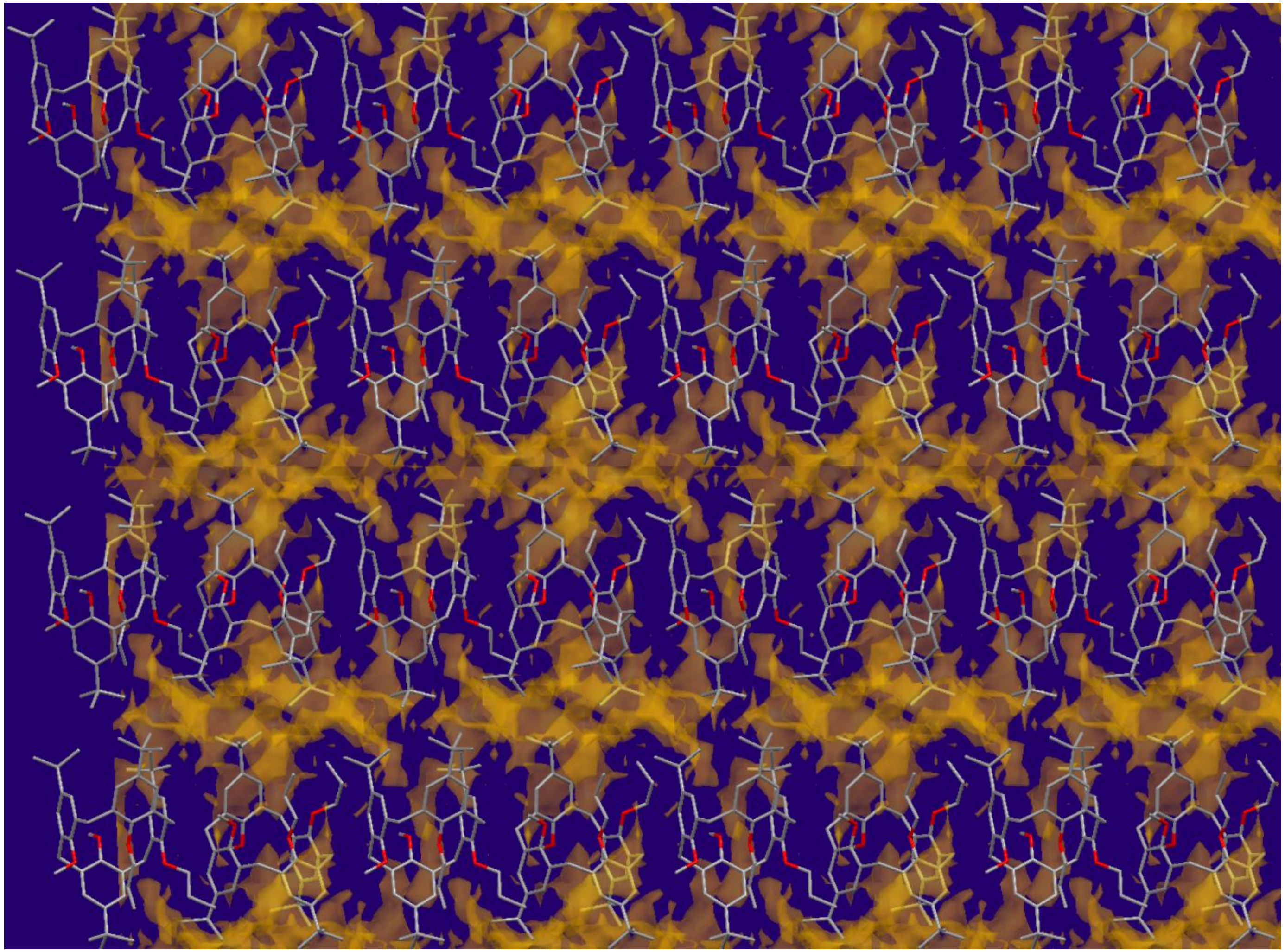

Figure S11: Packing motif of structure **12** along the crystallographic *c*-direction. The yellow areas denote solvent accessible empty voids.
